# The risk of new-onset atrial fibrillation in patients with type 2 diabetes mellitus treated with sodium glucose cotransporter 2 inhibitors versus dipeptidyl peptidase-4 inhibitors

**DOI:** 10.1186/s12933-020-01162-w

**Published:** 2020-11-06

**Authors:** Ann Wan-Chin Ling, Cze-Ci Chan, Shao-Wei Chen, Yi-Wei Kao, Chien-Ying Huang, Yi-Hsin Chan, Pao-Hsien Chu

**Affiliations:** 1 Cardiovascular Department, Chang Gung Memorial Hospital, Linkou, Taoyuan 33305 Taiwan; 2 Division of Thoracic and Cardiovascular Surgery, Department of Surgery, Chang Gung Memorial Hospital, Linkou Medical Center, Chang Gung University, Taoyuan City, Taiwan; 3 Center for Big Data Analytics and Statistics, Linkou Medical Center, Chang Gung Memorial Hospital, Taoyuan City, Taiwan; 4 Graduate Institute of Business Administration, College of Management, Fu Jen Catholic University, Taipei, Taiwan; 5 College of Medicine, Chang Gung University, Taoyuan, 33302 Taiwan; 6 Microscopy Core Laboratory, Chang Gung Memorial Hospital, Linkou, Taoyuan Taiwan

**Keywords:** Atrial fibrillation, Type 2 diabetes mellitus, Sodium glucose cotransporter-2 inhibitor, Dipeptidyl peptidase-4 inhibitor, Heart failure

## Abstract

**Background:**

Sodium glucose cotransporter 2 inhibitor (SGLT2i) reduces the risk of hard cardiovascular endpoints in type 2 diabetes mellitus (T2DM) patients with/without established cardiovascular diseases. Whether SGLT2i is associated with a lower risk of new-onset atrial fibrillation (AF) in T2DM patients is unclear. We aimed to evaluate the risk of new-onset AF associated with the use of SGLT2i compared to dipeptidyl peptidase-4 inhibitor (DPP4i) among a longitudinal cohort of diabetic patients.

**Methods:**

We used medical data from a multi-center healthcare provider in Taiwan, which included a total of 15,606 and 12,383 patients treated with SGLT2i and DPP4i, respectively, from June 1, 2016 to December 31, 2018. We used propensity-score weighting to balance covariates across study groups. Patients were followed up from the drug index date until the occurrence of new-onset AF, discontinuation of the index drug, or the end of the study period, whichever occurred first.

**Results:**

Overall, 55%, 45%, and 0% of the patients were treated with empagliflozin, dapagliflozin, and canagliflozin, respectively. Most patients in the DPP4i group were prescribed with linagliptin (51%), followed by sitagliptin (24%), saxagliptin (13%), vildagliptin (8%) and alogliptin (5%). The use of SGLT2i was associated with a lower risk of new-onset AF compared with DPP4i after propensity-score weighting [hazard ratio: 0.61; 95% confidential interval: 0.50–0.73; *P* < 0.001]. Subgroup analysis revealed that the use of SGLT2i was associated with a lower risk of new-onset AF compared with DPP4i across several subgroups including old age, female in gender, the presence of cardiovascular disease, hemoglobin A1c $\ge$ 8%, and chronic kidney disease. The advantage of SGLT2i over DPP4i persisted with different SGLT2i (dapagliflozin or empagliflozin) and either low- or standard-dose SGLT2i.

**Conclusions:**

SGLT2i was associated with a lower risk of new-onset AF compared with DPP4i among T2DM patients in real-world practice.

## Background

Atrial fibrillation (AF) is the most common sustained cardiac arrhythmia worldwide, and it is associated with higher risks of ischemic stroke, heart failure hospitalization and mortality [1–3]. Diabetes mellitus (DM) is associated with higher risks of ischemic cardiovascular events, heart failure event irrespective to ischemic event, and mortality, and also a 40% higher risk of AF in the general population [4–7]. In patients with DM and established cardiovascular disease, those with AF at baseline had a higher risk of worse heart failure outcomes than those without AF [8, 9]. Pathophysiological mechanisms including atrial electrical, structural, autonomic remodeling, oxidative stress, inflammation, and glycemic fluctuations have been suggested to explain the association between DM and occurrence of AF. [10, 11] Sodium-glucose cotransporter 2 inhibitor (SGLT2i) is a new class of anti-diabetic drug which inhibit sodium and glucose reabsorption in proximal tubules of the kidney and thereby lower blood glucose in patients with type 2 diabetes mellitus (T2DM) [12]. Three randomized placebo controlled trials have shown that SGLT2i (including canagliflozin, dapagliflozin, and empagliflozin) reduced the risk of hard cardiovascular endpoints in T2DM patients with/without established cardiovascular diseases [13–15]. SGLT2i has been shown to have multiple pleiotropic effects of glucose-independent and direct cardiac protection, including mitigating inflammation, oxidative stress, endothelial dysfunction, and left ventricular dysfunction, which may improve atrial remodeling and thus reduce the risk of AF [16, 17]. Although these randomized controlled trials have shown firm evidence of the benefits of SGLT2i with regards to ischemic cardiovascular diseases and mortality, all-cause mortality, and heart failure hospitalizations in patients with a high cardiovascular risk, whether SGLT2i themselves reduce the risk of atrial arrhythmia or AF is unclear. Recently, the post-hoc analysis of the DECLARE-TIMI 58 trial indicated that dapagliflozin significantly reduced the risk of AF/atrial flutter (AFL) in T2DM patients [18]. However, the CVD-REAL Nordic showed no significant difference of the new-onset AF associated with the use of dapagliflozin compared with dipeptidyl peptidase-4 inhibitor (DPP4i) or other glucose-lowering agents in T2DM patients in real-world practice [19, 20]. Therefore, the primary aim of the present study was to investigate whether SGLT2i is associated with a decreased risk of incident AF compared with DPP4i, specifically focused on Asian population with T2DM, in a large real-world setting.

## Methods

### Database

The study was based in part on data from the Chang Gung Research Database provided by Chang Gung Memorial Hospital (CGMH). The interpretation and conclusions contained herein do not represent the position of Chang Gung Memorial Hospital (CGMH). This study was approved by the Institutional Review Board of the Chang Gung Medical Foundation. We conducted this retrospective observational study using patient data from the CGMH Medical System. The CGMH Medical System is composed of two medical center, two regional hospitals, and three district hospitals with a total of 10,050 beds and around 280,000 admissions per year, and it is currently the largest healthcare provider in Taiwan.[21, 22] The advantage of the CGMH medical database is that detailed data on diagnoses, interventions, medications, laboratory examinations, and imaging are available for each patient. [22, 23] The identification number of each patient is encrypted and de-identified using a consistent encryption procedure; therefore, the need for informed consent was waived for this study.

### Study design and outcome

**Fig. 1:**
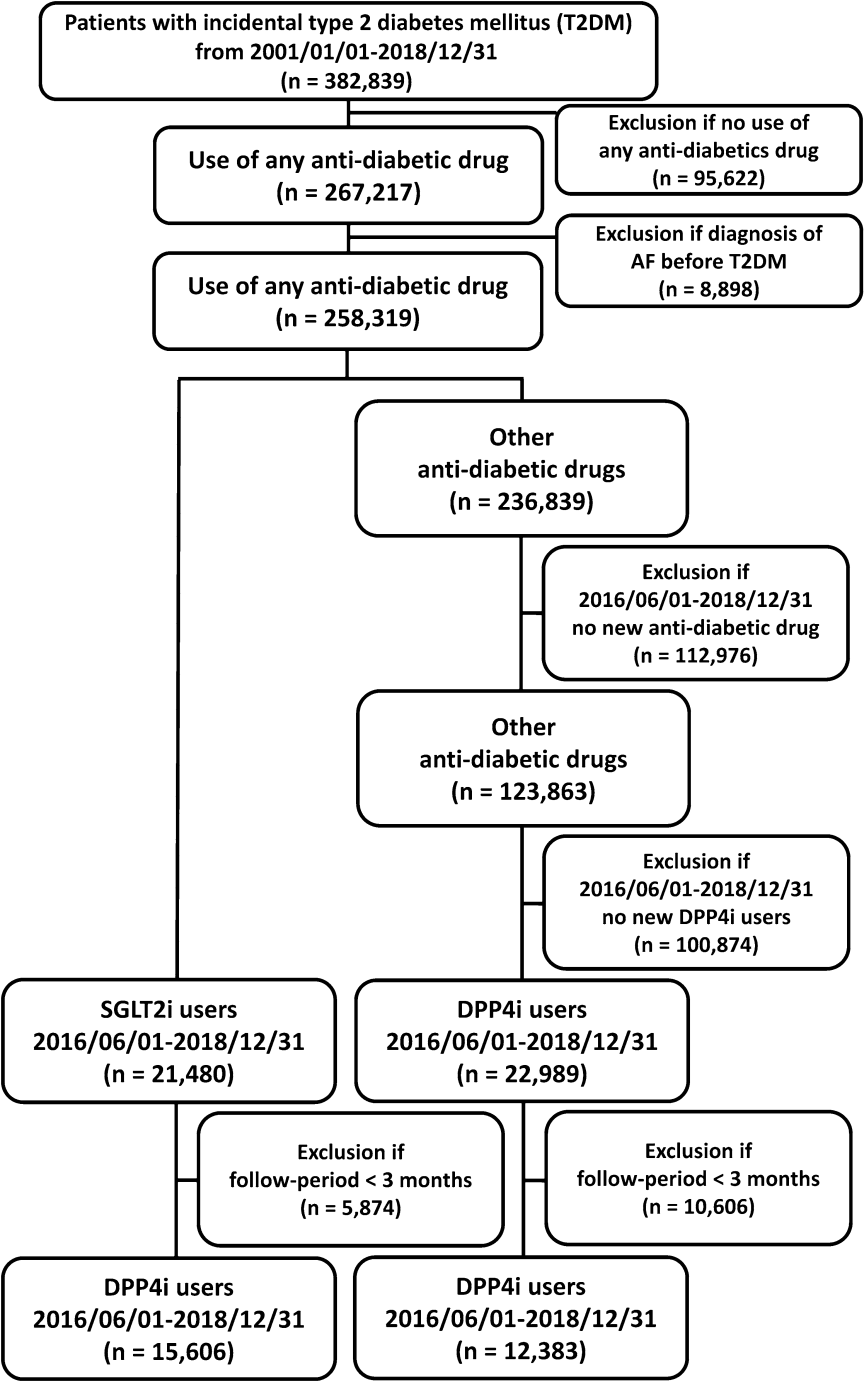
Enrollment of patients with type 2 diabetes mellitus (T2DM) treated with sodium glucose cotransporter 2 inhibitors (SGLT2i) versus dipeptidyl peptidase-4 inhibitors (DPP4i). A total of 15,606 T2DM patients treated with SGLT2i were compared with 12,383 patients treated with DPP4i from June 1, 2016 to December 31, 2018. AF = atrial fibrillation; DPP4i = dipeptidyl peptidase-4 inhibitor; SGLT2i = sodium glucose cotransporter 2 inhibitor; T2DM = type 2 diabetes mellitus

The flowchart of the study design and patient enrollment is shown in Fig. 1. The CGMH Research Database was retrospectively searched for patients $\ge$ 20 years of age in whom new-onset T2DM was diagnosed from January 1, 2001 to December 31, 2018 (n = 382,839). Patients who did not use any ant-diabetic drugs (n = 95,622) were excluded from the present study. We also excluded patients with a diagnosis of AF before a diagnosis of T2DM (n = 8898). Among the 258,319 patients treated with any anti-diabetic drug without a diagnosis of AF, those who had a first prescription for a SGLT2i (approval date: June 1, 2016) were enrolled in the present study (n = 21,480). Of the other 236,839 patients who received other non-SGLT2i treatments, 22,989 had a first prescription for a DPP4i after June 1, 2016. We restricted the study patients by only considering those patients with a minimal follow-up period of more than 3 months in the CGMH Medical System. Finally, there were 15,606 and 12,383 patients treated with SGLT2i and DPP4i, respectively, with a minimal following-up period of $\ge$ 3 months enrolled in the present study. Among those 15,606 SGLT2i user, there were 7376 (47%) patients with a previous history of DPP4i exposure. The ATC codes of SGLT2i and DPP4i in the present study were summarized in Additional file 1: Table S1. The study outcome was defined as the diagnosis of new-onset AF (International Classification of Diseases, Ninth Revision, Clinical Modification [ICD-9-CM] code 427.31 from January 1, 2010, to December 31, 2015, and ICD-10-CM code I48 from January 1, 2016, to December 31, 2018) in at least one inpatient or outpatient department visit occurred at least 1 month after the drug index date. In addition, we also validated the ICD codes for identifying AF by analyzing the 5276 12-lead ECG indicating AF in the inpatient or outpatient claims database of CGMH between 2015 and 2018. Among them, there were 4908 ECG had the ICD diagnosis indicating AF. Therefore, the positive predictive value of AF coding in CGMH Medical System was 93.03%. For each group, the index date was defined as the first date of a prescription for a SGLT2i or DPP4i after June 1, 2016. The follow-up period was defined as the period from the index date until the occurrence of new-onset AF, discontinuation of the index drug, mortality, the latest follow-up date in the CGMH Medical System, or the end of the study period (December 31, 2018), whichever occurred first.

### Covariates

Baseline characteristics referred to any claims record with the above diagnoses or medication codes prior to the drug index date. The ischemic etiology of the T2DM patients was defined by one of the following criteria: 1) ≥ 75% luminal diameter stenosis of the main epicardial coronary artery; 2) history of myocardial infarction or coronary revascularization; and 3) myocardial ischemia or infarction documented in myocardial perfusion imaging. A history of any prescription medicine was confined to medications taken at least once within 3 months preceding the index date. Baseline laboratory data listed in Table 1 were based on the measurements performed within 1 year before the drug index date.

**Table 1:** Clinical characteristics of the patients with type 2 diabetes treated with SGLT2i and DPP4i before and after inverse probability of treatment weights (IPTW)

	Before IPTW			After IPTW		
	SGLT2i (n = 15,606)	DPP4i (n = 12,383)	ASMD	SGLT2i (n = 27,054.45)	DPP4i (n = 26,889.05)	ASMD
Clinical characteristics						
Diabetes duration	2310.07 ± 1316.40	1509.97 ± 1452.10	0.577	1966.89 ± 1397.89	1917.41 ± 1497.30	0.034
Age (year)	58.52 ± 11.76	62.52 ± 125.88	0.045	59.63 ± 12.03	60.43 ± 134.37	0.008
Female	6515 (41.7)	5472 (44.2)	0.049	11,861.4 (43.1)	11,712.7 (43.6)	0.009
Ischemic heart etiology	1482 (9.5)	824 (6.7)	0.104	2256.3 (8.2)	2095.9 (7.8)	0.015
Hypertension	10,350 (66.3)	6527 (52.7)	0.280	16,781.5 (61.0)	16,036.0 (59.6)	0.028
Dyslipidemia	11,315 (72.5)	5732 (46.3)	0.554	16,808.6 (61.1)	15,912.7 (59.2)	0.039
Cerebral vascular accidents	782 (5.0)	1004 (8.1)	0.125	1680.4 (6.1)	1759.2 (6.5)	0.018
Congestive heart failure	587 (3.8)	488 (3.9)	0.009	1026.6 (3.7)	1038.0 (3.9)	0.007
Chronic lung disease	322 (2.1)	376 (3.0)	0.062	645.0 (2.3)	680.1 (2.5)	0.012
Chronic liver disease	3980 (25.5)	2175 (17.6)	0.194	6093.8 (22.2)	5720.7 (21.3)	0.021
Chronic kidney disease	2639 (16.9)	2417 (19.5)	0.068	4770.4 (17.3)	4801.1 (17.9)	0.013
Peripheral artery disease	137 (0.9)	144 (1.2)	0.028	270.4 (1.0)	300.4 (1.1)	0.013
Gout	1506 (9.7)	1152 (9.3)	0.012	2664.5 (9.7)	2627.1 (9.8)	0.003
Malignancy	1146 (7.3)	1401 (11.3)	0.137	2441.2 (8.9)	2525.0 (9.4)	0.018
Vital sign						
Height (cm)	161.95 ± 12.62	160.26 ± 12.44	0.135	161.15 ± 13.05	160.97 ± 12.11	0.014
Body weight (KG)	74.37 ± 15.54	68.08 ± 39.50	0.210	71.87 ± 15.20	72.02 ± 81.04	0.003
BMI	28.05 ± 4.85	26.29 ± 15.89	0.150	27.40 ± 4.85	27.59 ± 33.11	0.008
SBP (mmHg)	139.10 ± 19.66	139.29 ± 21.62	0.009	139.21 ± 20.28	139.23 ± 20.94	0.001
DBP (mmHg)	78.18 ± 11.83	76.83 ± 12.62	0.110	77.79 ± 11.85	77.53 ± 12.52	0.021
HR (bpm)	84.73 ± 13.44	83.83 ± 14.45	0.065	84.44 ± 13.69	84.29 ± 14.42	0.010
Baseline laboratory data						
HbA1c (%)	8.86 ± 1.67	8.37 ± 1.97	0.270	8.68 ± 1.66	8.60 ± 2.02	0.041
eGFR (ml/min/m^2^)	94.28 ± 31.15	77.07 ± 41.83	0.467	89.64 ± 31.06	87.96 ± 47.23	0.042
ALT (U/L)	34.47 ± 38.40	31.67 ± 38.24	0.073	33.44 ± 35.24	33.47 ± 43.42	0.001
Triglycerides (mg/dL)	186.24 ± 250.65	169.85 ± 171.68	0.076	179.63 ± 217.31	180.47 ± 254.64	0.004
LDL (mg/dL)	94.56 ± 30.90	98.49 ± 33.64	0.122	97.02 ± 32.78	96.89 ± 32.39	0.004
HDL (mg/d)	43.56 ± 11.09	43.22 ± 12.12	0.029	43.68 ± 11.26	43.55 ± 12.10	0.011
Baseline medications						
Anti-platelet agent	5298 (33.9)	3501 (28.3)	0.123	8565.2 (31.1)	8258.3 (30.7)	0.009
Statin	9455 (60.6)	5166 (41.7)	0.384	14,457.3 (52.6)	13,587.2 (50.5)	0.041
Non-dihydropyridine CCB	811 (5.2)	580 (4.7)	0.024	1471.4 (5.3)	1435.1 (5.3)	0.001
Dihydropyridine CCB	2555 (16.4)	2669 (21.6)	0.132	5071.6 (18.4)	5033.0 (18.7)	0.007
Beta-blocker	5248 (33.6)	3431 (27.7)	0.129	8624.8 (31.4)	8197.4 (30.5)	0.019
ACEI or ARB or ARNI	9448 (60.5)	6035 (48.7)	0.239	15,511.8 (56.4)	14,791.4 (55.0)	0.028
MRA	462 (3.0)	377 (3.0)	0.005	816.8 (3.0)	818.4 (3.0)	0.004
Loop diuretics	1058 (6.8)	1344 (10.9)	0.144	2168.5 (7.9)	2317.4 (8.6)	0.027
Nitrate	988 (6.3)	723 (5.8)	0.021	1668.9 (6.1)	1586.9 (5.9)	0.007
Digoxin	104 (0.7)	64 (0.5)	0.020	157.8 (0.6)	157.9 (0.6)	0.002
Anti-diabetic agent						
SU	10,342 (66.3)	5033 (40.6)	0.532	15,197.2 (55.3)	14,296.7 (53.2)	0.042
Metformin	14,011 (89.8)	8224 (66.4)	0.589	22,258.8 (80.9)	21,131.2 (78.6)	0.058
Glinide	479 (3.1)	796 (6.4)	0.158	1220.9 (4.4)	1276.1 (4.7)	0.015
Glitazone	3826 (24.5)	690 (5.6)	0.550	4602.4 (16.7)	3701.6 (13.8)	0.083
Acarbose	3077 (19.7)	1017 (8.2)	0.337	4130.1 (15.0)	3567.1 (13.3)	0.050
Insulin	2560 (16.4)	2152 (17.4)	0.026	4465.3 (16.2)	4437.0 (16.5)	0.007

*ACEI* angiotensin-converting enzyme inhibitor, *ALT* alanine aminotransferase, *ARB* angiotensin receptor blocker, *ARNI* angiotensin receptor-neprilysin inhibitor, *BMI* body mass index, *CCB* calcium channel blocker, *DBP* diastolic blood pressure, *DPP4i* dipeptidyl peptidase-4 inhibitor, *eGFR* estimated glomerular filtration rate, *HBA1c* hemoglobin A1c, *HDL* high density lipoprotein, *HR* heart rate, *IPTW* inverse probability of treatment weights, *LDL* low density lipoprotein, *MRA* mineralocorticoid receptor antagonist, *SBP* systolic blood pressure, *SGLT2i* sodium glucose co-transporter-2 inhibitor, *SU* sulfonylurea

Data are expressed as mean ± standard deviation or as percentage %

### Statistical analysis

We used the propensity score method to simulate the effect of a randomized clinical trial for observational cohort data and to estimate the study outcomes of study groups [15]. The inverse probability of treatment weights (IPTW) of propensity scores was used to balance covariates across the four groups. The weights were derived to obtain estimates representing average treatment effects in the treated. All of the covariates listed in Table 1 were included in the propensity models. Incidence rates were estimated using the total number of study outcomes during the follow-up period divided by person-years at risk. The risk of time-dependent study outcomes for two study groups was obtained using survival analysis (Kaplan–Meier method and log-rank test and Cox proportional hazards regression). The balance of covariates at baseline among the study groups was assessed using the absolute standardized mean difference (ASMD) rather than statistical testing, because balance is a property of the sample and not of an underlying population. Another advantage of using ASMD is that it is not influenced by sample size. An ASMD value ≤ 0.1 was defined as indicating a negligible difference in potential confounders between two study groups (Table 1). Statistical significance was defined as a *P-*value < 0.05. All statistical analyses were performed using SAS 9.4 (SAS Institute Inc., Cary, NC, USA).

## Results

A total of 15,606 SGLT2i users and 12,383 DPP4i users were eligible for the study. The median following-up periods for the SGLT2i and DPP4i groups were 1.48 [0.81–2.10] and 1.05 [0.60–1.67] years, respectively. Among the SGLT2i users, 8525 (55%), 7035 (45%), and 46 (0%) were treated with empagliflozin, dapagliflozin, and canagliflozin, respectively. Most of the DPP4i users were prescribed with linagliptin (n = 6287, 51%), followed by sitagliptin (n = 2929, 24%), saxagliptin (n = 1636, 13%), vildagliptin (n = 972, 8%), and alogliptin (n = 559, 5%).

Table 1 summarizes the baseline demographic characteristics, comorbidities, and medications of the two groups. Before propensity score weighting, the SGLT2i group had a longer DM duration, higher prevalence rates of ischemic heart disease, hypertension, dyslipidemia and chronic liver disease, and lower prevalence rates of stroke history and diagnosed cancer. The SGLT2i group had higher serum hemoglobin A1c (HbA1c) and estimated glomerular filtration rate (eGFR) than the DPP4i group. For baseline medications, the SGT2i group had a higher prescription rate of anti-platelet agents, statins, angiotensin-converting enzyme inhibitors/angiotensin receptor blockers and concomitant anti-diabetic agents including sulfonylureas, metformin, and glitazone than the DPP4i group (ASMD > 0.1). After propensity-score weighting, the two study groups were well-balanced in all characteristics (all ASMD < 0.1).

**Fig. 2:**
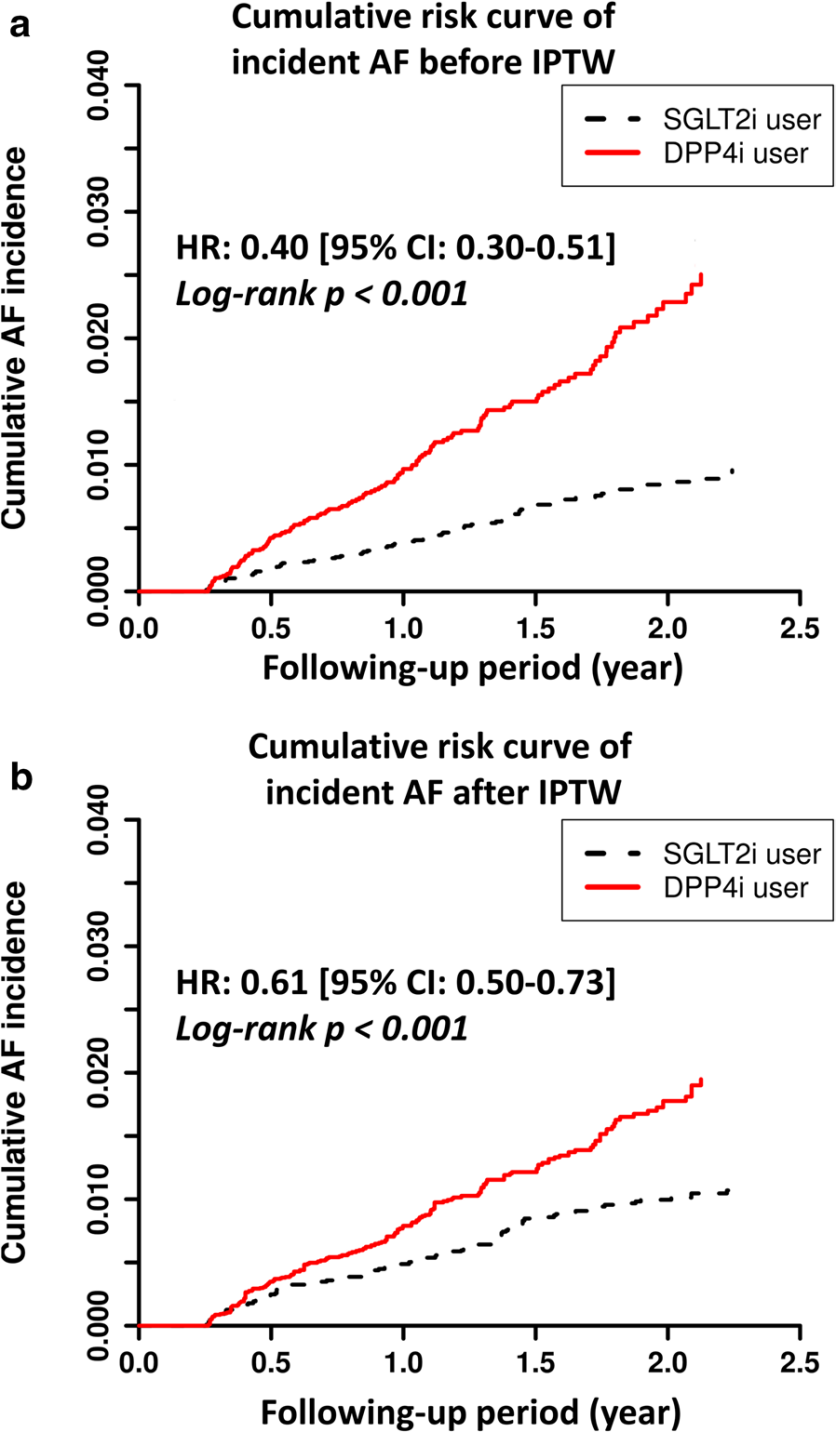
Cumulative risk curve of incident atrial fibrillation (AF) for the study cohorts treated with SGTL2i versus DPP4i before and after inverse probability of treatment weights (IPTW). SGLT2i showed a significantly lower cumulative risk of new-onset AF compared with DPP4i in T2DM patients before and after IPTW. aHR = adjusted hazard ratio; CI = confidential interval; IPTW = inverse probability of treatment weights. Other abbreviations as in Fig. 1

**Fig. 3:**
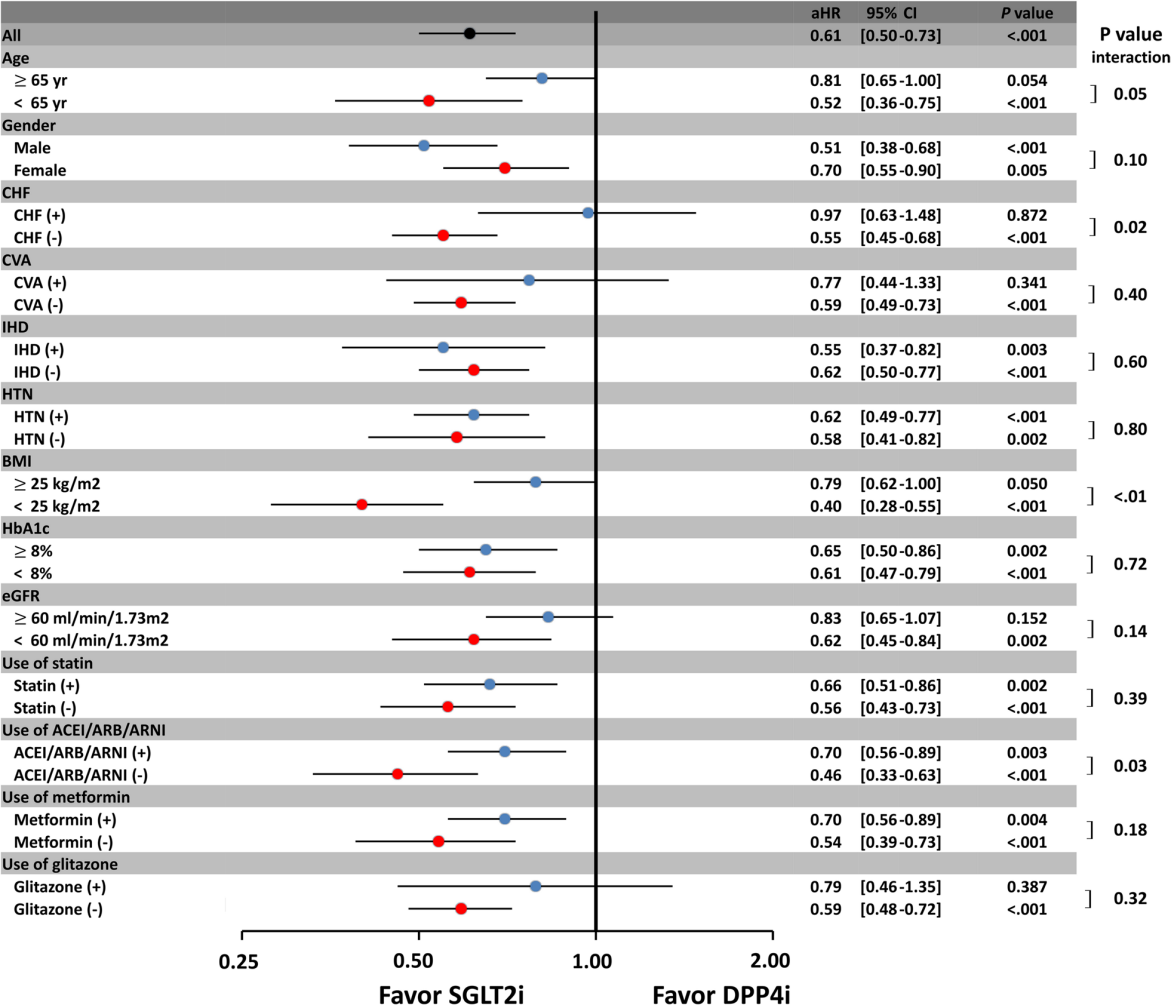
Subgroup analysis of forest plot of hazard ratio (HR) for SGLT2i versus DPP4i among T2DM patients after IPTW. Subgroup analysis showed consistent results for a lower risk of incident AF for SGLT2i vs. DPP4i among T2DM patients aged $\ge$ 65 years, female in gender, and those with cerebral vascular disease (CVA), ischemic heart disease (IHD), hypertension (HTN), hemoglobin A1c (HbA1c) $\ge$ 8%, estimated glomerular filtration rate (eGFR) < 60 ml/min/1.73 m^2^, and the use of concomitant medications as the main analysis (*P* interaction > 0.05). Of note, the use of SGLT2i reduced the number of new-onset AF events in subgroups including those without previous history of heart failure, those with BMI < 25 kg/m^2^, and those without concomitant use of renin-angiotensin system blockers (*P* interaction < 0.05). ACEI = angiotensin-converting enzyme inhibitor; ARB = angiotensin receptor blocker; ARNI = angiotensin receptor-neprilysin inhibitor; BMI = body mass index; CHF = congestive heart failure; CVA = cerebral vascular disease; eGFR = estimated glomerular filtration rate; HbA1c = hemoglobin A1c; HTN = hypertension; IHD = ischemic heart disease. Other abbreviations as in Figs. 1 and 2

There were 93 and 146 new-onset AF event occurred in the SGLT2i and DPP4i groups, respectively, during the following-up period. For a total of 93 AF outcome in SGLT2i group, there were 51 and 42 events recorded in the outpatient and inpatient service, respectively. For a total of 146 AF outcome in SGLT2i group, there were 42 and 104 events recorded in the outpatient and inpatient service, respectively. The SGLT2i users were associated with a lower risk of new-onset AF compared with the DPP4i users, both before and after propensity-score weighting (hazard ratio (HR): 0.61; [95% confidential interval (CI) 0.50–0.73]; *P* < 0.001). There was a clear separation of event curves for new-onset AF between these two groups both before and after propensity score weighting adjustments (Fig. 2). Subgroup analysis revealed that the use of SGLT2i was associated with a lower risk of new-onset AF compared with the use of DPP4i across most subgroups (Fig. 3). Furthermore, the use of SGLT2i was associated with greater reductions in new-onset AF events in subgroups including those without previous history of heart failure, those with a BMI of < 25 kg/m^2^, and those without concomitant use of renin-angiotensin system blockers (*P* interaction < 0.05).

**Fig. 4:**
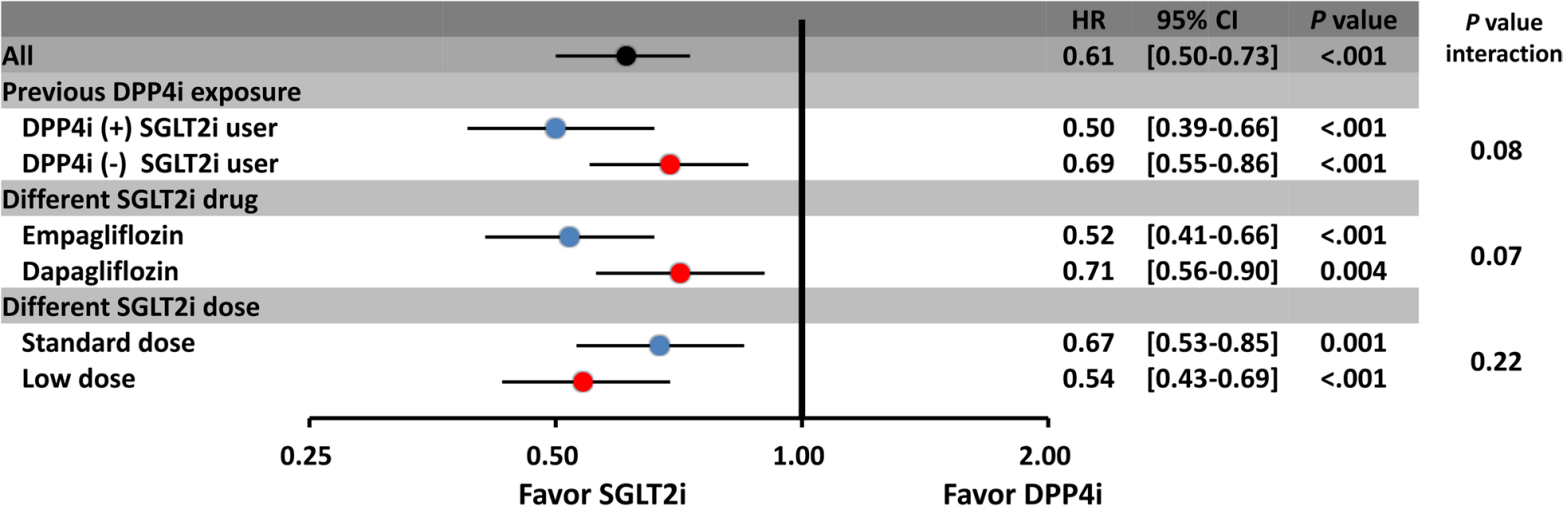
Subgroup analysis of forest plot of HR for SGLT2i versus DPP4i among T2DM patients with/without previous DPP4i exposure, treated with different SGLT2i or different SGLT2i dosages after IPTW. The benefits of SGLT2i over DPP4i in lowering the risk of incident AF persisted with previous DPP4i-exposure SGTL2i user, different SGLT2i (dapagliflozin or empagliflozin) and both low- (empagliflozin 10 mg or dapagliflozin 5 mg once daily) and standard-dose (empagliflozin 25 mg or dapagliflozin 10 mg once daily) SGLT2i treatment. The abbreviations as in Fig. 3

Among those 15,606 SGLT2i user, there were 7376 (47%) and 8230 (53%) patients with and without a previous history of DPP4i exposure. Both the DPP4i-epxerinced (HR: 0.50; [95% CI 0.39–0.66]; *P* < 0.001) and DPP4i-naïve (HR: 0.69; [95% CI 0.55–0.86]; *P* < 0.001) SGLT2i users were both associated with a lower risk of new-onset AF compared to DPP4i users (*P* interaction > 0.05). In addition, the advantage of SGLT2i over DPP4i in lowering the risk of incident AF persisted with different SGLT2i (dapagliflozin or empagliflozin) and both low-dose (empagliflozin 10 mg or dapagliflozin 5 mg once daily) and standard-dose (empagliflozin 25 mg or dapagliflozin 10 mg once daily) SGLT2i treatment (Fig. 4).

### Sensitivity test

Sensitivity analyses were performed by using a propensity score matching (PSM) model to test if the results were still consistent with the main analysis by using IPTW. We used 1:1 PSM to balance covariates across the two study groups. There were 7233 paired patients treated with SGLT2i and DPP4i enrolled in the analysis after PSM, and all covariates were well-balanced after PSM (ASMD all < 0.1) (Additional file 1: Table S2). The use of SGLT2i was associated with a lower risk of new-onset AF than DPP4i after PSM (HR: 0.68; [95% CI 0.48–0.99]; *P* = 0.043) consistent with the main analysis. We also used the standardized mortality ratio weighting (SMRW) [24] rather than the IPTW to test the consistency of our study result. The use of SGLT2i was associated with a lower risk of new-onset AF than DPP4i after SMRT (HR: 0.63; [95% CI 0.48–0.83]; *P* = 0.001) consistent with the main analysis. It is possible that some severe diabetic patients died before AF can occur. Therefore, the HR for risk of new-onset AF between the two study groups were analyzed after IPTW and using death as a competing risk factor. The use of SGLT2i was associated with a lower risk of new-onset AF than DPP4i after IPTW and using death as an competing factor (HR: 0.61; [95% CI 0.51–0.74]; *P* < 0.001) consistent with the main analysis.

## Discussion

To the best of our knowledge, this is the largest observational study to specifically evaluate the risk of new-onset AF focused on Asian population with T2DM treated with SGLT2i versus DPP4i. Our results showed that the use of SGLT2i was associated with a significantly lower risk of new-onset AF compared to DPP4i among T2DM patients. The benefits in reducing the risk of new-onset AF with SGLT2i over DPP4i persisted in several important subgroups including old age, presence of cardiovascular disease, impaired renal function, and elevated HbA1c levels. In addition, the advantages of SGLT2i over DPP4i in lowering the risk of new-onset AF persisted with different SGLT2i drugs (dapagliflozin or empagliflozin) and both low and standard doses of SGLT2i.

T2DM or insulin resistance is an important risk factor for ischemic stroke and the development of new-onset AF [25, 26]. An animal study demonstrated that diabetic rat atria had greater interstitial fibrosis, lower connexin 40 expression, and decreased conduction velocity. In addition, the diabetic atria showed electrical remodeling with prolongation of action potential duration (APD), an increase in spatial dispersion and frequency-dependent shortening of APD, and increased incidence of APD alternans [27]. All of these factors facilitated the formation of re-entry associated atrial arrhythmia. Other studies have also reported adrenergic activation and heterogeneous sympathetic innervation in diabetic hearts, suggesting that neural remodeling may play a crucial role in diabetes-related atrial arrhythmia [28]. Furthermore, T2DM itself is associated with several chronic diseases including obesity, hypertension, chronic kidney disease, and heart failure, all of which further increase the risk of incident AF [6, 7, 29, 30].

SGLT2i is a new class of anti-hyperglycemic agents that inhibit glucose absorption by the proximal tubules of the kidney, resulting in glycosuria [31]. SGTL2i has been shown to reduce blood sugar levels, blood pressure, body weight, albuminuria, lipid profile, arterial stiffness, and endothelial function via an insulin-independent mechanism in T2DM patients [32]. Recent study indicated that SGLT2i had more favorable pleiotropic effects on body weight, liver function and eGFR changes when compared to DPP4i, potentially modifying the cardio-metabolic disease risks in T2DM patients [33]. Moreover, SGLT2i has been shown to have impressive cardioprotective and renoprotective effects. The main mechanisms of their cardioprotective effects are improvements in cardiac cell metabolism and ventricular loading conditions, inhibition of Na^+^/H^+^ exchange in myocardial cells, alterations in adipokine and cytokine production, and reductions in cardiac cell necrosis and cardiac fibrosis [34, 35]. SGLT2i has also been shown to reduce sympathetic overdrive, which plays an important role in the development of AF [18].

Other diabetes medications including metformin, thiazolidinedione (TZD), and DPP4i, may also be associated with a lower risk of AF. A previous study of a nationwide, population-based dynamic cohort indicated that the use of metformin was associated with a decreased risk of AF in T2DM patients who were not using other antidiabetic drugs, probably by attenuating atrial cell tachycardia-induced myolysis and oxidative stress [36]. Another study indicated that the use of DPP4i as second-line antidiabetic drugs was associated with a lower risk of AF compared with other second-line antidiabetic drugs among T2DM patients treated with metformin in real-world practice [37]. TZD is an insulin sensitizer that also have anti-inflammatory and anti-oxidative effects, and they might decrease the risk of AF compared with other antidiabetic drugs. *Pallisgaard *et al*.* reported that the use of TZD was associated with a 24% reduction in the risk of incident AF compared with other antidiabetic drugs as second-line treatment among T2DM patients [38]. However, no significant differences in the risk of incident AF with use of TZD were reported in the PROactive, RECORD, and BARI 2D trials [39–41].

Three large randomized controlled trials, EMPA-REG OUTCOME (empagliflozin), CANVAS Program (canagliflozin), and DECLARE–TIMI 58 (dapagliflozin) demonstrated that three SGLT2i significantly reduced the risk of heart failure hospitalization in T2DM patients with/without established cardiovascular diseases compared with the current standard-of-care diabetes management [13–15]. Furthermore, the DAPA-HF trial indicated that dapagliflozin treatment reduced the risk of worsening heart failure or cardiovascular death by 26% compared to placebo among patients with heart failure and a reduced ejection fraction of < 40%, regardless of the presence or absence of T2DM [42]. However, despite the potential improvements in atrial remodeling mediated by SGLT2i, few clinical studies have investigated the relationship between the use of SGLT2i and the risk of AF, and the results have been inconsistent. A meta-analysis of 35 eligible randomized controlled trials (canagliflozin, nine; empagliflozin, eight; dapagliflozin, 18), showed that SGLT2i significantly reduced all-cause mortality, major adverse cardiac events, non-fatal myocardial infarction, and heart failure hospitalization in T2DM patients compared to placebo. However, no significant difference was noted in the occurrence of stroke, unstable angina, or AF (odd ratio: 0.61; [95% CI 0.31–1.19]; *P* = 0.15) [43]. The CVD-REAL Nordic study also indicated that dapagliflozin was associated with lower risks of cardiovascular events and all-cause mortality but a neutral risk of AF (HR: 0.92; [95% CI 0.76–1.12]; P = 0.414) compared with DPP-4is in a real-world clinical setting [19, 20]. Conversely, post-hoc analysis of the DECLARE-TIMI 58 trial indicated that dapagliflozin reduced the risk of AF/AFL by 19% (HR: 0.81; [95% CI 0.68–0.95]; *P* = 0.009) and the number of total AF/AFL events by 23% compared to placebo in 17,160 T2DM patients, regardless of the presence or absence of AF/AFL, established cardiovascular disease, or heart failure at baseline [18]. To the best of our knowledge, only the CVD‐REAL Nordic study has directly compared the risk of AF between SGLT2i and DPP4i treatment among T2DM patients in a real-world setting [19]. In contrast to the CVD‐REAL Nordic study, reporting a comparable risk of new-onset AF, we report a lower risk associated with SGLT2i compared with DPP4i treatment. This finding is of particular interest because it is more in line with the results of post-hoc analysis of the DECLARE-TIMI 58 trial. The seemingly discrepant findings between our results and those of the CVD‐REAL Nordic study could be attributable to chance and/or the presence of different baseline characteristics including ethnicity, the prevalence of underlying chronic kidney disease or AF (9% patients already have AF at baseline in the CVD‐REAL Nordic study), and a different drug prescription including the use of insulin, glitazone, and glucagon-like peptide-1 receptor agonist. The CVD-REAL Nordic study used a large National claim database but with a lacking of several important data including laboratory data and vital sign, which was also different from our study using a large multicenter hospital-based *electronic medical record* database with those important data available. Nevertheless, whether the study outcomes in our present study were confounded by those factors cannot not be excluded and need further elucidation. Further prospective and randomized studies are necessary to clarify our results.

## Study limitations

We choose the DPP4i as an active comparator because it is a relatively new and widely used anti-hypoglycemic agent until now. Also, several important studies have used the DPP4i as the comparator [19, 33, 44, 45]. There are several limitations to the present study. First, we did not have serial ECG data to help identify whether the patients diagnosed with AF had persistent or paroxysmal AF related to acute illnesses such as hyperthyroidism or infection. Moreover, we lacked data of other unmeasured confounding factors such as the physicians’ choice of medications, use of tobacco or alcohol, race, and family history. Second, long-term outcome comparisons such as 5 or 10 years of follow-up were not included in this study as SGLT2i is a relatively new drug compared to other antidiabetic drugs. Third, this was a retrospective and observational study. The clinical characteristics of the patients were different across SGLT2i and DPP4i groups. Although we adjusted for several important parameters relevant to clinical outcomes by using propensity score weighting models, residual unmeasured confounders were still probably present. We suggest that future prospective randomized studies are needed to determine whether our findings are applicable to T2DM patients. Fourth, the advantage of CGMH medical database is that each patient’s detailed medical activity is all available in the database. However, the CGMH datasets is a closed medical system without external link to protect each patient’s privacy in CGMH database. Therefore, we cannot obtain data from outside the CGMH database in Taiwan, which may have resulted in loss to follow-up or underestimation of medical activity for each patient outside the CGMH system. This important limitation should be kept in mind when interpreting the results we presented here [46]. Fifth, our study was performed in an on-treatment design**,** and did not take the changes of medical status or activity (e.g. new diagnosis of co-morbidities, eGFR decline, discontinuation/add-on of co-medication) during their following-up period which may result in different outcome of patients into considerations. Sixth, we did not analyze the relative risk of AF for canagliflozin versus DPP4i in the subgroup analysis due to a very limited number of patients (n = 46, 0.29%) and short follow-up period (approved after March 1, 2018) in the present study. Further studies are needed to investigate whether the treatment benefits of empagliflozin and dapagliflozin in lowering the risk of new-onset AF can be extrapolated to canagliflozin. Lastly, we only investigated Asian patients, and whether our results can be extrapolated to other races remains unclear.

## Conclusions

The use of SGLT2i was associated with a lower risk of incident AF compared DPP4i among T2DM patients, irrespective of underlying comorbidities or different SGLT2i in a large real-world setting.

## Supplementary information

**Additional file 1: Table S1.** The ATC code of SGLT2i and DPP4i. **Table S2.** Clinical characteristics of the patients with type 2 diabetes treated with SGLT2i and DPP4i before and after 1:1 propensity score matching (PSM).

## Data Availability

The datasets used and/or analyzed in the current study are available from the corresponding author on reasonable request.

## Abbreviations

ACEI: Angiotensin-converting enzyme inhibitor
AF: Atrial fibrillation
ACEI: Angiotensin-converting enzyme inhibitor
ALT: Alanine aminotransferase
ARB: Angiotensin receptor blocker
ARNI: Angiotensin receptor-neprilysin inhibitor
ASMD: Absolute standardized mean difference
AMI: Acute myocardial infarction
APT: Antiplatelet agent
BMI: Body mass index
CKD: Chronic kidney disease
DBP: Diastolic blood pressure
DM: Diabetes mellitus
DPP4i: Dipeptidyl peptidase-4 inhibitor
eGFR: Estimated glomerular filtration rate
HbA1c: Hemoglobin A1c
HDL: High-density lipoprotein
IHD: Ischemic heart disease
IPTW: Inverse probability of treatment weights
LDL: Low-density lipoprotein
MRA: Mineralocorticoid Receptor Antagonist
PAD: Peripheral artery disease
PSM: Propensity score matching
SBP: Systolic blood pressure
SGLT2i: Sodium glucose cotransporter-2 inhibitor
SU: Sulfonylurea
T2DM: Type 2 diabetes mellitus
