# Supplementary material for: The risk of new-onset atrial fibrillation in patients with type 2 diabetes mellitus treated with sodium glucose cotransporter 2 inhibitors versus dipeptidyl peptidase-4 inhibitors
Source: Cardiovasc Diabetol. 2020 Nov 6;19:188. doi: 10.1186/s12933-020-01162-w (PMC7648323; doi:10.1186/s12933-020-01162-w)
Supplement: Supplementary file 1 — Additional file 1: Table S1. The ATC code of SGLT2i and DPP4i. Table S2. Clinical characteristics of the patients with type 2 diabetes treated with SGLT2i and DPP4i before and after 1:1 propensity score matching (PSM). [file 12933_2020_1162_MOESM1_ESM.docx]

**Table S1** The ATC code of SGLT2i and DPP4i

| **SGLT2i** |  |
| --- | --- |
| Empagliflozin | A10BK03 |
| Dapagliflozin | A10BK01 |
| Canagliflozin | A10BK02 |
| Empagliflozin + Metformin | A10BD20 |
| Dapagliflozin + Metformin | A10BD15 |
| **DPP4i** |  |
| Alogliptin | A10BH04 |
| Linagliptin | A10BH05 |
| Sitagliptin | A10BH01 |
| Saxagliptin | A10BH03 |
| Vildagliptin | A10BH02 |
| Alogliptin + Metformin | A10BD13 |
| Linagliptin + Metformin | A10BD11 |
| Sitagliptin + Metformin | A10BD07 |
| Saxagliptin + Metformin | A10BD10 |
| Vildagliptin + Metformin | A10BD08 |

DPP4i = dipeptidyl peptidase-4 inhibitor; SGLT2i = sodium glucose co-transporter-2 inhibitor

**Table S2** Clinical characteristics of the patients with type 2 diabetes treated with SGLT2i and DPP4i before and after 1:1 propensity score matching (PSM)

|  | **Before PSM** | | | **After PSM** | | |
| --- | --- | --- | --- | --- | --- | --- |
|  | **SGLT2i**  **(n = 15606)** | **DPP4i**  **(n = 12383)** | **ASMD** | **SGLT2i**  **(n = 7233)** | **DPP4i**  **(n = 7233)** | **ASMD** |
|  | **Clinical characteristics** | | | | | |
| **Diabetes duration** | 2310.07 ±1316.40 | 1509.97 ±1452.10 | 0.577 | 1898.73 ±1368.04 | 1882.72 ±1458.44 | 0.011 |
| **Age (yr)** | 58.52 ±11.76 | 62.52 ±125.88 | 0.045 | 59.85 (11.81) | 59.14 (164.30) | 0.006 |
| **Female** | 6515 (41.7) | 5472 (44.2) | 0.049 | 3188 (44.1) | 3177 (43.9) | 0.003 |
| **Ischemic heart etiology** | 1482 (9.5) | 824 (6.7) | 0.104 | 558 (7.7) | 572 (7.9) | 0.007 |
| **Hypertension** | 10350 (66.3) | 6527 (52.7) | 0.280 | 4340 (60.0) | 4341 (60.0) | <0.001 |
| **Dyslipidemia** | 11315 (72.5) | 5732 (46.3) | 0.554 | 4375 (60.5) | 4374 (60.5) | <0.001 |
| **Cerebral vascular accidents** | 782 (5.0) | 1004 (8.1) | 0.125 | 494 (6.8) | 496 (6.9) | 0.001 |
| **Congestive heart failure** | 587 (3.8) | 488 (3.9) | 0.009 | 274 (3.8) | 269 (3.7) | 0.004 |
| **Chronic lung disease** | 322 (2.1) | 376 (3.0) | 0.062 | 183 (2.5) | 181 (2.5) | 0.002 |
| **Chronic liver disease** | 3980 (25.5) | 2175 (17.6) | 0.194 | 1542 (21.3) | 1558 (21.5) | 0.005 |
| **Chronic kidney disease** | 2639 (16.9) | 2417 (19.5) | 0.068 | 1266 (17.5) | 1243 (17.2) | 0.008 |
| **Peripheral artery disease** | 137 (0.9) | 144 (1.2) | 0.028 | 77 (1.1) | 72 (1.0) | 0.007 |
| **Gout** | 1506 (9.7) | 1152 (9.3) | 0.012 | 718 (9.9) | 682 (9.4) | 0.017 |
| **Malignancy** | 1146 (7.3) | 1401 (11.3) | 0.137 | 704 (9.7) | 677 (9.4) | 0.013 |
|  | **Vital sign** | | | | | |
| **Height (cm)** | 161.95 ±12.62 | 160.26 ±12.44 | 0.135 | 160.94 ±12.81 | 160.93 ±12.09 | 0.001 |
| **Body weight (KG)** | 74.37 ±15.54 | 68.08 ±39.50 | 0.210 | 70.94 ±13.63 | 70.65 ±50.53 | 0.008 |
| **BMI** | 28.05 ±4.85 | 26.29 ±15.89 | 0.150 | 27.11 ±4.36 | 27.06 ±20.48 | 0.004 |
| **SBP (mmHg)** | 139.10 ±19.66 | 139.29 ±21.62 | 0.009 | 138.89 ±19.99 | 138.94 ±20.38 | 0.002 |
| **DBP (mmHg)** | 78.18 ±11.83 | 76.83 ±12.62 | 0.110 | 77.60 ±11.75 | 77.76 ±12.42 | 0.013 |
| **HR (bpm)** | 84.73 ±13.44 | 83.83 ±14.45 | 0.065 | 84.19 ±13.52 | 84.18 ±14.31 | 0.001 |
|  | **Baseline laboratory data** | | | | | |
| **HbA1c (%)** | 8.86 ±1.67 | 8.37 ±1.97 | 0.270 | 8.65 ±1.64 | 8.66 ±2.03 | 0.010 |
| **Estimated GFR (ml/min/m^2^)** | 94.28 ±31.15 | 77.07 ±41.83 | 0.467 | 88.66 ±28.79 | 89.34 ±41.15 | 0.019 |
| **ALT (U/L)** | 34.47 ±38.40 | 31.67 ±38.24 | 0.073 | 33.16 ±28.24 | 33.65 ±39.10 | 0.015 |
| **Triglycerides (mg/dL)** | 186.24 ±250.65 | 169.85 ±171.68 | 0.076 | 177.00 ±193.80 | 175.95 ±197.07 | 0.005 |
| **LDL (mg/dL)** | 94.56 ±30.90 | 98.49 ±33.64 | 0.122 | 97.08 ±31.86 | 97.35 ±32.42 | 0.008 |
| **HDL (mg/d)** | 43.56 ±11.09 | 43.22 ±12.12 | 0.029 | 43.90 ±11.34 | 43.92 ±12.02 | 0.002 |
|  | **Baseline medications** | | | | | |
| **Anti-platelet agent** | 5298 (33.9) | 3501 (28.3) | 0.123 | 2192 (30.3) | 2210 (30.6) | 0.005 |
| **Statin** | 9455 (60.6) | 5166 (41.7) | 0.384 | 3746 (51.8) | 3769 (52.1) | 0.006 |
| **Non-dihydropyridine CCB** | 811 (5.2) | 580 (4.7) | 0.024 | 373 (5.2) | 358 (4.9) | 0.009 |
| **Dihydropyridine CCB** | 2555 (16.4) | 2669 (21.6) | 0.132 | 1327 (18.3) | 1359 (18.8) | 0.011 |
| **Beta-blocker** | 5248 (33.6) | 3431 (27.7) | 0.129 | 2154 (29.8) | 2216 (30.6) | 0.019 |
| **ACEI or ARB or ARNI** | 9448 (60.5) | 6035 (48.7) | 0.239 | 3965 (54.8) | 3989 (55.2) | 0.007 |
| **MRA** | 462 (3.0) | 377 (3.0) | 0.005 | 219 (3.0) | 200 (2.8) | 0.016 |
| **Loop diuretics** | 1058 (6.8) | 1344 (10.9) | 0.144 | 583 (8.1) | 535 (7.4) | 0.025 |
| **Nitrate** | 988 (6.3) | 723 (5.8) | 0.021 | 419 (5.8) | 409 (5.7) | 0.006 |
| **Digoxin** | 104 (0.7) | 64 (0.5) | 0.020 | 48 (0.7) | 42 (0.6) | 0.011 |
| **Anti-diabetic agent** |  |  |  |  |  |  |
| **SU** | 10342 (66.3) | 5033 (40.6) | 0.532 | 3780 (52.3) | 3834 (53.0) | 0.015 |
| **Metformin** | 14011 (89.8) | 8224 (66.4) | 0.589 | 5981 (82.7) | 6063 (83.8) | 0.030 |
| **Glinide** | 479 (3.1) | 796 (6.4) | 0.158 | 304 (4.2) | 303 (4.2) | 0.001 |
| **Glitazone** | 3826 (24.5) | 690 (5.6) | 0.550 | 668 (9.2) | 642 (8.9) | 0.013 |
| **Acarbose** | 3077 (19.7) | 1017 (8.2) | 0.337 | 841 (11.6) | 827 (11.4) | 0.006 |
| **Insulin** | 2560 (16.4) | 2152 (17.4) | 0.026 | 1181 (16.3) | 1164 (16.1) | 0.006 |

ACEI = angiotensin-converting enzyme inhibitor; ALT = alanine aminotransferase; ARB = angiotensin receptor blocker; ARNI = angiotensin receptor-neprilysin inhibitor; BMI = body mass index; CCB = calcium channel blocker; DBP = diastolic blood pressure; DPP4i = dipeptidyl peptidase-4 inhibitor; eGFR = estimated glomerular filtration rate; HBA1c = hemoglobin A1c; HDL = high density lipoprotein; HR = heart rate; LDL = low density lipoprotein; MRA = mineralocorticoid receptor antagonist; PSM = propensity score matching; SBP = systolic blood pressure; SGLT2i = sodium glucose co-transporter-2 inhibitor; SU = sulfonylurea

Data are expressed as mean ± standard deviation or as percentage %.
